# Biomonitoring PhIP, a Potential Prostatic Carcinogen, in the Hair of Healthy Men of African and European Ancestry

**DOI:** 10.3390/toxics13010042

**Published:** 2025-01-08

**Authors:** Robert J. Turesky, Clarence Jones, Jingshu Guo, Kari Cammerrer, Laura A. Maertens, Emmanuel S. Antonarakis, Zhanni Lu, Logan G. Spector

**Affiliations:** 1 Masonic Cancer Center, Department of Medicinal Chemistry, University of Minnesota, Minneapolis, MN 55455, USA; jingshu.guo@thermofisher.com (J.G.); camme004@umn.edu (K.C.); 2 Hue-Man Partnership, 2400 Park Ave., Minneapolis, MN 55404, USA; clarencejones7428@gmail.com; 3 Clinical Research and Toxicology, Chromatography and Mass Spectrometry Division, Thermo Fisher Scientific, 355 River Oaks Parkway, San Jose, CA 95134, USA; 4 Masonic Cancer Center, Division of Environmental Health Sciences, School of Public Health, University of Minnesota, Minneapolis, MN 55455, USA; maert006@umn.edu; 5 Department of Medicine, University of Minnesota, Minneapolis, MN 55455, USA; anton401@umn.edu; 6 Masonic Cancer Center, Division of Pediatric Epidemiology and Clinical Research, University of Minnesota, Minneapolis, MN 55455, USA; luxxx371@umn.edu

**Keywords:** biomarkers, carcinogens, cooked meat, hair dosimeter, heterocyclic aromatic amines, PhIP, prostate cancer

## Abstract

Heterocyclic aromatic amines (HAAs), formed during the cooking of meat, are potential human carcinogens, underscoring the need for long-lived biomarkers to assess exposure and cancer risk. Frequent consumption of well-done meats containing 2-amino-1-methyl-6-phenylimidazo[4,5-*b*]pyridine (PhIP), a prevalent HAA that is a prostatic carcinogen in rodents and DNA-damaging agent in human prostate cells, has been linked to aggressive prostate cancer (PC) pathology. African American (AA) men face nearly twice the risk for developing and dying from PC compared to White men. We previously demonstrated that scalp hair is a reliable biospecimen for measuring PhIP intake using liquid chromatography-mass spectrometry. This study aimed to determine whether PhIP dietary intake is higher in AA men, potentially contributing to this health disparity. Healthy AA men were found to have a significantly higher mean hair PhIP level (2.12-fold) than White men on free-choice diets. However, this difference was not statistically significant after adjusting for melanin content. Further research is needed to understand how hair pigmentation, follicular density, and other morphological features of hair influence PhIP accumulation. These insights can improve the accuracy of using hair PhIP levels as a biomarker for exposure and its potential associations with cancer risk.

## 1. Introduction

More than 25 genotoxic heterocyclic aromatic amines (HAAs) form in cooked meats, poultry, and fish [1]. HAAs induce cancer at multiple sites in laboratory animals [2]. The International Agency for Research on Cancer (IARC) recently classified red meat as a Group 2A carcinogen (probably carcinogenic to humans), identifying the colorectum, pancreas, and the prostate gland as target organs based on epidemiological data and mechanistic studies [3]. Prostate cancer (PC) is the second leading cause of cancer-related death among men in the United States; however, the chemicals contributing to PC risk remain uncertain and require further study [4,5]. Several epidemiological studies have linked frequent consumption of well-done cooked red meat to risk of aggressive PC pathology, while eating rare or medium-cooked meat does not show this association [6,7,8,9]. 2-Amino-1-methyl-6-phenylimidazo[4,5-*b*]pyridine (PhIP) is one of the most mass-abundant carcinogenic HAAs formed in well-done cooked meats [1]. Furthermore, PhIP stands out as the sole HAA formed in cooked meats that is known to induce PC in rodents [10,11]. PhIP is also a potent DNA-damaging agent in human prostate cells [12,13,14]. Thus, high-temperature cooking, which produces PhIP and other carcinogenic HAAs, may significantly influence PC development and progression.

The PC incidence and mortality rates are approximately twice as high in Black men compared to White men in the U.S. [5]. This disparity has persisted for many years and reflects the global burden of PC in Black men. Differences in healthcare access, early diagnosis, and germline and somatic genetics between races may contribute to this variability and the higher PC susceptibility in men of African ancestry [15,16]. Lifestyle factors, including a diet rich in well-done meat, may also play an important role in determining PC risk [6,7,8,9]. A U.S. national dietary survey reported that AA men consume significantly more well-done cooked meats, leading to a 2- to 3-fold higher PhIP intake than White men [17]. This finding aligns with a study conducted in Los Angeles, which showed higher levels of PhIP and its metabolites in the urine of AA men [18]. One hypothesis is that the preference for well-done meat may contribute to an increased risk of PC and more aggressive disease in Black men. However, the association between red meat consumption and PC risk is controversial, as some studies have not found significant associations [19,20,21].

A critical limitation in many epidemiology studies is their reliance on Food Frequency Questionnaire (FFQs) tools. While FFQs are useful for estimating an individual’s common food and nutrient intake and assessing dietary patterns, they do not provide quantitative measurements of dietary intake of potential carcinogens. The reliance on the respondents’ memory and ability to recall also makes FFQs imprecise [22], particularly for quantitatively assessing HAA intake, where concentrations in cooked meats can vary by more than 100-fold [1,23]. This variability can lead to inaccurate estimates of the individuals’ exposure levels of HAAs and associated cancer risks. Therefore, validated biomarkers that capture and quantify long-term exposure are needed to accurately assess dietary intake and the health impact of HAAs [24,25,26].

**Figure 1:**
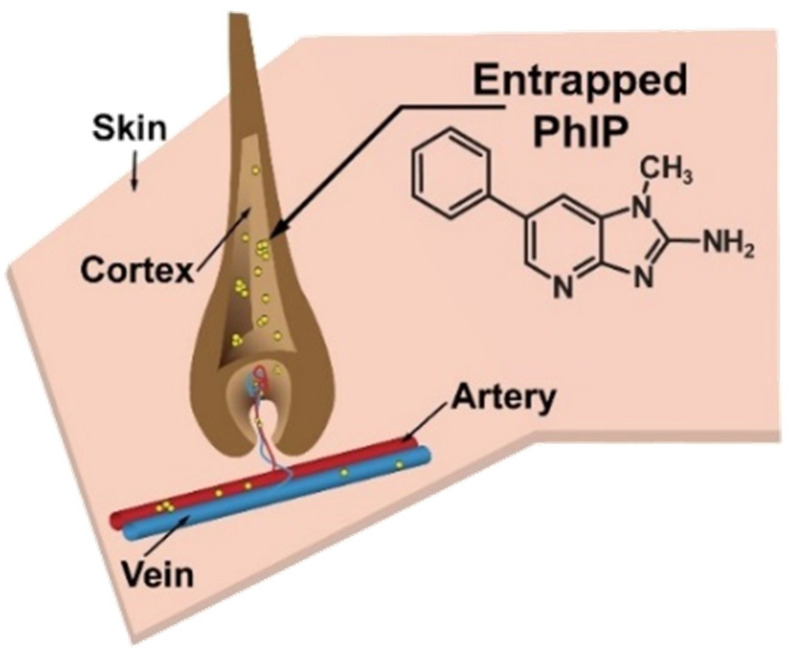
PhIP (shown as yellow balls) entrapment in hair follicles after first-pass metabolism.

Hair is a valuable non-invasive biospecimen for biomonitoring, as various chemicals accumulate in hair, and its extended growth cycle enables long-term measurement of an integrated exposure [27]. Alexander and colleagues demonstrated that a small portion of the dose of PhIP becomes entrapped within the melanin-rich tissues, including the follicles of rodent fur, and that PhIP becomes incorporated into the cortex of the newly grown fur shafts [28]. Recent studies have employed liquid chromatography-mass spectrometry (LC-MS) to quantify PhIP in human hair specimens [29], including from our laboratory (Figure 1) [30].

Our prior research demonstrated a strong correlation between PhIP intake from cooked meats and PhIP levels in hair, with newly grown hair from the nuchal/occipital region of the scalp capturing dietary intake over the preceding 10–12 weeks in volunteers on semi-controlled meat diets [31,32]. We later observed that hair PhIP levels were significantly higher in PC patients compared to those with bladder cancer or benign prostatic hyperplasia [33], supporting epidemiological findings that link well-done meat consumption with aggressive PC pathology [6,7,8,9]. We propose that the hair PhIP dosimeter may provide a more accurate assessment of dietary exposure to this procarcinogen, a proxy for well-done meat consumption, than FFQs. In this pilot study, we applied the hair PhIP dosimeter to healthy AA and White men on free-choice diets to investigate potential racial differences in dietary exposure to PhIP.

## 2. Materials and Methods

### 2.1. Study Population

Two cohorts of male participants were recruited. Inclusion criteria required that participants were healthy, 17 years of age or older, had natural hair color, and followed an omnivorous diet without restrictions. Smokers were included in the study. Exclusion criteria specified that participants must not have used hair dyes, hair straighteners, or hair cremes other than typical shampoos within the past two years. Potential volunteers were provided background information on the purpose of the study; those who agreed to participate signed an informed consent form. The first cohort was recruited at the Minnesota State Fair held in Minneapolis, 26–28 August 2017, and consisted predominantly of non-Hispanic White (NHW) men. Each participant completed a questionnaire module on environmental chemical exposures, which included questions regarding the frequency of meat consumption and cooking methods adapted from Dietary Questionnaire Study II (National Institutes of Health) (https://epi.grants.cancer.gov/dhq2, accessed on 26 November 2024), and the American Cancer Society’s Cancer Prevention Study 3 [34]. Our research staff collected newly grown hair (50–100 mg) from the nuchal/occipital region of the scalp, near the hairline using electric clippers. Hair samples were placed in Ziploc plastic bags, stored in envelopes to protect against light, and kept at −20 °C until analysis.

The second cohort, comprised of men from the African American (AA) community in greater Minneapolis, was recruited from 28 August to 8 September 2023, through the Hue-MAN Partnership, a coalition of organizations and individuals dedicated to addressing public health issues important to the AA community. Volunteers had their hair cut from the nuchal/occipital region of the scalp at their local barbershops, with hair samples collected and stored as described above. The AA men completed an abbreviated questionnaire on environmental exposures, focusing on tobacco and alcohol use and meat and seafood consumption, accompanied by photographs illustrating meat doneness preferences, depicting internal doneness and external brownness [35].

### 2.2. Description of Demographic Information and Meat Consumption

All hair specimens were blind-coded with no identifying information. Demographic information analyzed included: age, race/ethnicity, body mass index (BMI), and various lifestyle factors, such as smoking history and alcohol consumption (one drink defined as a 12 ounce beer, 5 ounce glass of wine, or 1.5 shots of liquor). Meat consumption data included frequencies of charred/barbecued meat consumption in the past seven days, types of meat consumed, and preferred cooking methods. Meat types were classified as charred/barbecued red meats, poultry, seafood, or processed meats. A charred barbecued food index was calculated from the participants’ consumption frequencies, with higher scores reflecting more frequent charred/barbecued meats and seafood intake.

### 2.3. Chemicals and Reagents

PhIP and 1-[^2^H_3_C]-PhIP were purchased from Toronto Research Chemicals (Toronto, ON, Canada). Waters Oasis MCX cartridges (1 cc cartridges containing 30 mg resin) were from Waters (New Milford, MA, USA). LC-MS grade water, formic acid (HCO_2_H), methanol, and acetonitrile (CH_3_CN) were from Fisher Chemicals (Pittsburgh, PA, USA). Soluene 350 was from PerkinElmer (Waltham, MA, USA). All other reagents, unless specified, were ACS grade. Cap LC vials Chromacol 03-FISV were from Thermo Scientific (Waltham, MA, USA).

### 2.4. Chemical Analysis of Hair

#### 2.4.1. PhIP Isolation from Hair and Mass Spectrometric Measurements

Hair samples (25 mg) were minced with an electric clipper and then placed in 2 mL Eppendorf tubes. The samples were rinsed with 0.1N HCl by vortexing, followed by a methanol rinse to remove any external chemical deposits on the hair surface. Afterward, the hair samples were air-dried in a ventilated hood. The hair was spiked with 1-[^2^H_3_C]-PhIP (500 pg per g hair) and digested by hydrolysis in 1N NaOH (1 mL) at 80 °C for 1 h. The PhIP was isolated by solvent extraction with ethyl acetate followed by solid phase extraction [30,31]. The eluent was concentrated to dryness by vacuum centrifugation. The extracts were reconstituted in 0.2 mL of 0.1% HCO_2_H and partitioned against 0.2 mL of methylene dichloride. The aqueous phase was vacuum centrifuged to dryness in the capLC vial and reconstituted in 25 µL 89.9% H_2_O/10% CH_3_CN/0.1% HCO_2_H.

PhIP measurements were carried out with an Orbitrap Lumos Tribrid MS interfaced to an UltiMate 3000 RSLCnano UHPLC system equipped with an Easy-Spray™ source (Thermo Fisher Scientific, San Jose, CA, USA). A Prontosil C18-AQ capillary column, 0.1 × 150 mm, 3 µm particle size, 100 Å pore size (Michrom, Auburn, CA, USA), was used for chromatography. The solvent A was 0.01% HCO_2_H, and solvent B was 95% CH_3_CN containing 0.01% HCO_2_H and 4.99% H_2_O. The injection volume was 1 µL. The solvent composition was held isocratic at 5% B for 4 min at a flow rate of 1 µL per min. Then, the solvent mixture increased linearly to 30% B at 18 min and then reached 95% B at 20 min, and it was held for 2 min. The column was equilibrated back to starting conditions over 2 min and held for 6 min before another injection commenced.

Data were acquired by Xcalibur version 4.4. MS parameters were as follows: polarity, positive ion; spray voltage, 2400 V; ion transfer tube temperature, 275 °C; RF lens 60%; quadrupole isolation, 2 *m*/*z*. PhIP was measured at the MS^3^ scan stage. PhIP (*m*/*z* 225.2 ⟶ 210.1 ⟶ *m*/*z* 100–240) and 1-[^2^H_3_C]-PhIP (*m*/*z* 228.1 ⟶ 210.1 ⟶ *m*/*z* 100–250). The MS^2^ activation was collision-induced dissociation (CID); activation Q, 0.5; activation time 50 ms; collision energy, 37%. The MS^3^ activation type was high-energy collision-induced dissociation (HCD) energy, 55%; maximum injection time, 246 ms; 1 microscan. The Orbitrap resolution was 120,000 (full width at half maximum) at *m*/*z* 200; normalized automatic gain control (AGC) target, 100%; data type, profile mode.

The calibration curve of PhIP was constructed using naturally colored brown hair in which PhIP was not detected. The hair was spiked at seven calibrant levels: 0, 25, 100, 200, 400, 500, and 1000 pg/g hair [30,36]. The internal standard, 1-[^2^H_3_C]-PhIP, was added to hair at 500 pg/g hair. PhIP and 1-[^2^H_3_C]-PhIP were measured at the MS^3^ scan stage using the common product ion at *m*/*z* 141.0573, while the *m*/*z* 183.0791 served as a qualifier ion to assess peak purity within a 10 ppm mass tolerance. Full product ion mass spectra at the MS^3^ scan stage confirmed the identities of PhIP and 1-[^2^H_3_C]-PhIP [36]. The data were fitted to a straight line (area of response of PhIP/internal standard versus the amount of PhIP/internal standard) using ordinary least-squares with equal weightings, showing good linearity (25–1000 pg PhIP/g hair) with a goodness-of-fit regression value of *r*^2^ > 0.998. The limit of quantification is 25 pg/g hair [36,37].

#### 2.4.2. Spectrophotometric Characterization of Melanin in Hair

Hair (1–3 mg) was digested in Soluene 350:H_2_O (9:1 *v*/*v*, 1 mL) by heating at 95 °C for 1 h. After equilibrating to room temperature, the spectra were acquired with an Agilent 8453 model UV/VIS spectrophotometer (Santa Clara, CA, USA). The absorbance at 500 nm represents the total amount of melanin (eumelanin and pheomelanins), and the A_650_/A_500_ ratio reflects the proportion of eumelanin to total melanin in hair. The estimate of melanin was based on the absorbance of 100 μg/mL of melanin (eumelanin) corresponding to 0.99 at 500 nm [38].

### 2.5. Statistical Analysis

The relationships between charred or barbecued meat consumption and PhIP levels in hair of AA and NHW men were examined using descriptive analysis and Spearman’s correlation. Variables compared between the groups included age, BMI, smoking status (never, former smokers, current), alcohol use (never, former, current), frequencies of charred/barbecued red meat/poultry, seafood, and processed meat consumption (none, 1–4, 5–7, 8–22 times; none vs. any), the number of charred/barbecued meat types consumed, and the charred barbecued food index. PhIP levels in hair were expressed as the average PhIP per gram of hair (pg/g hair) and PhIP adjusted for melanin (ng/g melanin) and compared between groups with further analysis stratified by smoking status (current smokers vs. non-smokers) and alcohol use (non-drinkers; drinking less than weekly, drinking weekly or more). Categorical variables were reported as frequencies and percentages, and continuous variables as medians and interquartile ranges (IQR). Statistical differences between groups were assessed using the Wilcoxon rank sum test and Pearson’s Chi-squared/Fisher’s exact tests. The Kruskal–Wallis rank sum test was applied to examine differences in alcohol consumption between groups. Spearman’s correlations were calculated to assess weekly consumption of charred/barbecued red meat, poultry, seafood, and processed meat with PhIP levels in hair and adjusted for melanin. Spearman’s rank-order correlation coefficient values were reported. All statistical analyses were conducted in R version 4.2.2, with two-sided *p*-values < 0.05 deemed significant.

## 3. Results and Discussion

### 3.1. Demographics of the Cohorts

The demographic data for non-Hispanic White (NHW) men and African American (AA) men are summarized in Table 1. Five AA participants were excluded due to self-reported Hispanic/Latino ethnicity (N = 1), mixed race (N = 1), or hair dye use (N = 3); one non-Hispanic White (NHW) volunteer was excluded for being of Hispanic/Latino ethnicity (N = 1). Consequently, 45 of the 50 AA participants, and 52 of the 53 White participants completed the demographics questionnaire and were successfully assayed for hair PhIP levels. The average ages of the cohorts differed significantly, with AA men having a median age of 47.0 years, compared to 60.2 years for White men (*p* = 0.032). The BMI values were not significantly different between the cohorts (*p* = 0.34). There were significant differences in lifestyle factors between the cohorts. Thirty-eight percent of AA men were current smokers (mean cigarettes per day and 95% CI (10, CI: 6.2, 13.8), compared to only 3.8% of the White cohort (*p* < 0.001). Thirty-one percent of AA men were current alcohol drinkers compared to 75% of the White men who drank alcohol weekly (*p* < 0.001).

**Figure 2:**
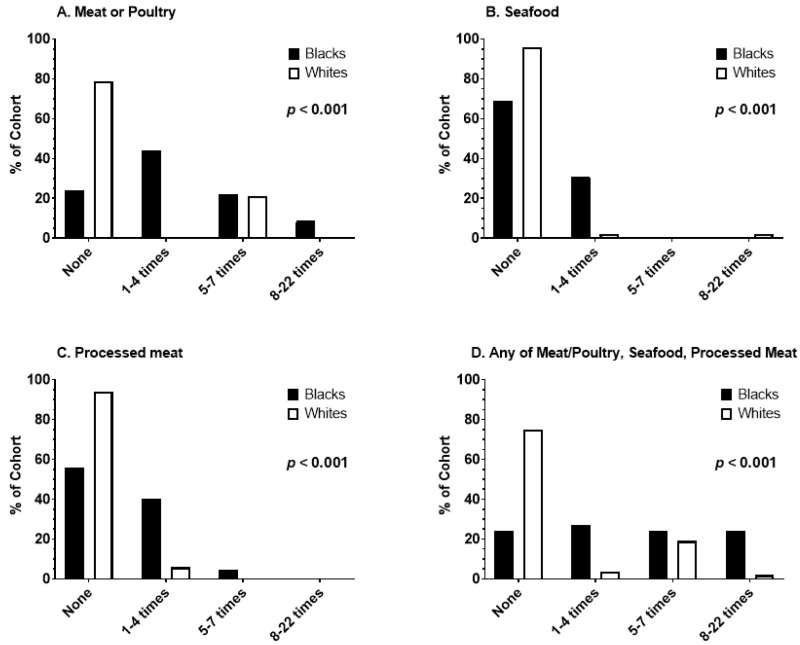
Frequencies of charred/barbecued meat consumption in the past week are shown. Panel (**A**) displays percentages (*y*-axis) of AA and NHW study participants consuming red meat or poultry 1–4, 5–7, 8–22 times, or none. Panels (**B**,**C**) show percentages for seafood and processed meat, respectively. Panel (**D**) summarizes percentages for any red meat or poultry, seafood, and processed meat consumption at the same frequencies.

Figure 2 and Supplementary Table S1 summarize the patterns of meat consumption and cooking preferences between the AA and White male cohorts. AA men ate charred barbecued meats and poultry with far greater frequency than White men (*p* < 0.001), as well as barbecued processed meats and seafood (*p* < 0.001). Among AA men, 76% reported eating charred/barbecued meat or poultry once or more times per week compared to 21% of the White men (*p* < 0.001). The composite measure of total weekly charred or barbecued meat consumption index scores were also significantly higher for AA men than for White men (*p* < 0.001).

### 3.2. PhIP Levels in Hair

Using hair as a biospecimen to assess PhIP exposure has several advantages over short-lived urinary or plasma biomarkers of PhIP. Many chemicals entrapped in the hair cortex, including PhIP, are stable and provide an integrated measure of exposure over time, while urinary and plasma biomarkers represent exposures occurring in the past 24–48 h only [25]. We sought to determine whether dietary meat patterns and a preference for well-done cooked meats could be distinguished between AA and White men by measuring PhIP levels in newly grown scalp hair, which captures the past 10–12 weeks of PhIP intake [31,32]. Hair samples were collected from White men in late August 2017, and from the AA cohort from late August to early September 2023, representing a six-year interval between the two cohorts. Thus, the hair specimens from both groups reflect PhIP intake during the summer, a season typically associated with barbequing and outdoor grilling in Minnesota.

**Figure 3:**
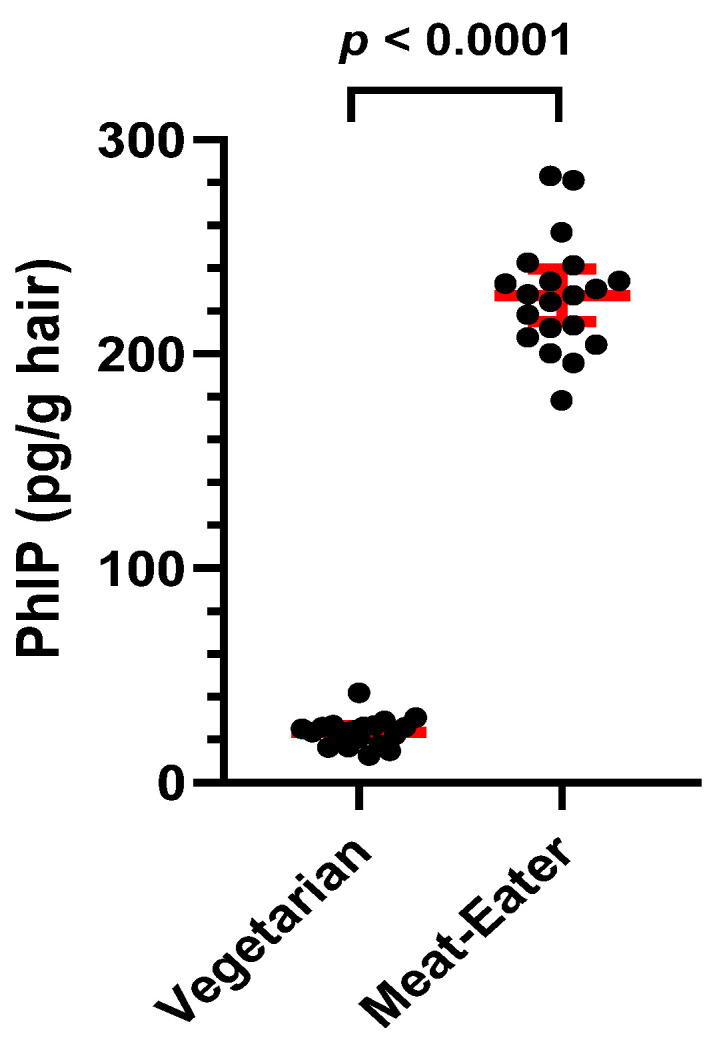
Scatter plot of quality control samples for PhIP analysis in hair samples from a vegetarian and meat-eater (mean and 95% CI).

PhIP is chemically stable in naturally colored brown and black hair samples, with levels measured within ±15% of the original values after storage at room temperature in the dark for up to 10 years (unpublished data, R. Turesky). However, as a precaution, hair samples from both cohorts were stored at −20 °C. Two quality control (QC) hair samples, which had been stored at room temperature for 10 years, were measured alongside hair samples from the AA and White cohorts. One QC hair sample was from a vegetarian, and the other QC sample was from an omnivore; both individuals were non-smokers. The mass spectrometry signal for PhIP in the vegetarian hair corresponded to 23.6 ± 6.4 pg PhIP/g hair (N = 20, mean ± SD), and the signal for PhIP in the omnivore was 227 ± 26 pg PhIP/g hair (N = 20), which was not statistically different from the original value obtained 10 years ago (Figure 3). All hair samples in both cohorts were assayed in duplicate (overall %RSD < 12%), and ten batches of hair samples were assayed over 3.5 months to complete the chemical analysis.

**Figure 4:**
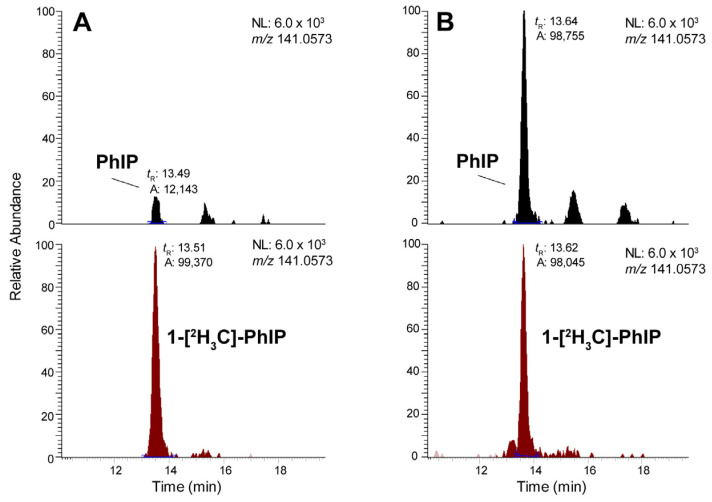
EIC of PhIP and 1-[^2^H_3_C]-PhIP in participants with (**A**) low (61 pg/g) and (**B**) high hair PhIP (504 pg/g) levels. Signal intensities are normalized to 1-[^2^H_3_C]-PhIP.

**Figure 5:**
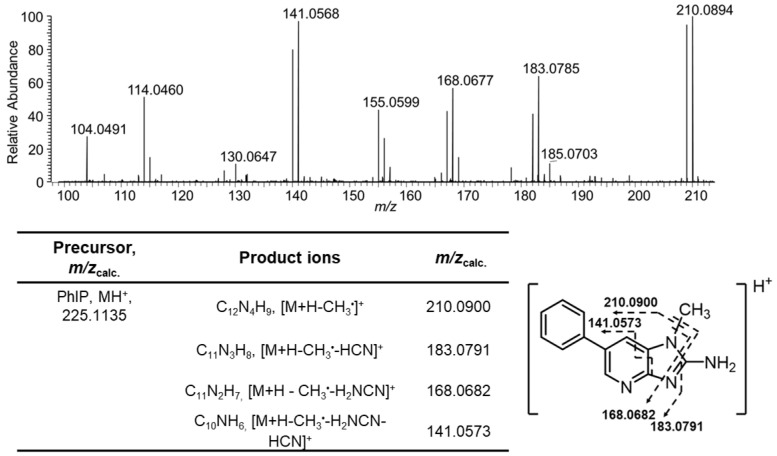
Mass spectrum of PhIP at the MS^3^ stage with Orbi trap detection at 120K resolution. The calculated *m*/*z* of the major fragment ions are shown in the table.

Representative extracted ion chromatograms (EIC) of PhIP and 1-[^2^H_3_C]-PhIP from participants with low and high hair PhIP levels are shown in Figure 4A,B, with the MS^3^ product ion spectrum of PhIP in the participant with high hair PhIP levels shown in Figure 5. The MS^3^ scan stage mass spectrum of 1-[^2^H_3_C]-PhIP is identical to PhIP [36].

### 3.3. The Influence of Melanin and Other Biological and Lifestyle Factors on Hair PhIP Levels

**Figure 6:**
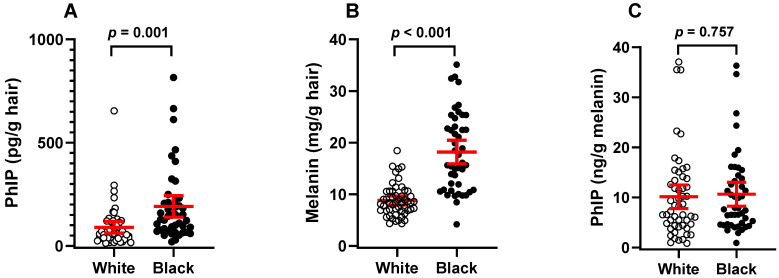
Scatter plot showing the mean and 95% CI for hair PhIP levels in AA and White men. (**A**) PhIP levels per g hair, (**B**) melanin content per g hair, and (**C**) PhIP levels adjusted per g melanin.

The PhIP hair levels and melanin content are summarized in Table 2 and depicted in Figure 6. The mean hair PhIP levels in AA men were 2.1-fold higher than in White men, suggesting a higher dietary PhIP intake in the AA cohort (Figure 6A). All 45 AA men had hair PhIP levels above the LOQ. In contrast, 7 of the 52 White men (13.5%) had hair PhIP levels below the LOQ. Fifteen White men (28.8%) had hair PhIP levels greater than 100 pg PhIP/g hair, compared to 28 AA men (62.2%); this level is 4-fold above the LOQ value. Additionally, 4 white men (7.7%) and 13 AA men (28.9%) had hair PhIP levels greater than 200 pg/g hair. These hair biomarker data align with the FFQ results, which reveal that AA men consumed more well-done charred/barbequed meat and seafood than White men.

PhIP binding to rodent and canine fur [39,40] and human hair [28,31] is influenced by pigmentation, with PhIP showing a high affinity for eumelanin. The mean melanin content in the hair of AA men is about 2.1 times greater than that of White men (*p* < 0.001) (Figure 6B), consistent with previous reports [41,42]. After adjusting the hair PhIP levels for melanin content, no statistically significant differences were observed between the AA and White cohorts (Figure 6C). Therefore, the hair PhIP biomarker may not reliably reflect differences in dietary PhIP intake between AA and White cohorts. While PhIP levels are readily distinguishable within each cohort when reported per unit hair weight or by melanin content, the differences narrow between the cohorts after adjusting for melanin.

Correlations between charred/barbecued meat consumption, hair PhIP levels, melanin content, PhIP levels in hair adjusted for melanin within AA and NHW men are reported in Supplementary Table S2. Among the AA men, charred/barbecued seafood consumption had a moderate and statistically significant correlation to PhIP levels in hair (ρ = 0.365, *p* = 0.0136), and this correlation was strengthened after hair PhIP levels were adjusted for melanin content (ρ = 0.398, *p* = 0.0067). While, in the NWH cohort, charred/barbecued seafood consumption was weakly, negatively correlated to PhIP levels in hair. This correlation was weaker when PhIP levels in hair were adjusted for melanin content. Additionally, the AA participants’ consumption of charred/barbecued red meat/poultry, seafood, and processed meat had significantly positive correlations with each other (red meat/poultry–seafood: ρ = 0.489, *p* < 0.0007; red meat/poultry/processed meat: ρ = 0.483, *p* < 0.0008; seafood–processed meat: ρ = 0.533, *p* < 0.0002). In the NHW men, no statistically significant correlations were observed between the consumption of these three types of charred/barbecued meats. However, the self-reported charred/barbecued meat/poultry consumption frequency did not show statistically significant correlations with PhIP levels in hair either without or with adjusting for melanin content, suggesting that the FFQ does not accurately capture dietary PhIP intake. This weak correlation between hair PhIP levels and weekly servings of red and processed meat, poultry, and fish is consistent with our previous study [33].

We validated the hair PhIP dosimeter in non-smoker volunteers, composed of nonsmoking Whites and Asian/Pacific Islanders, on a semi-controlled meat diet with known PhIP intake. Several factors influencing the accumulation of PhIP in hair were investigated [30,31,32]. Those studies demonstrated that PhIP levels in hair were correlated to PhIP intake (ρ = 0.53; *p* < 0.001), and this relationship was further strengthened when PhIP levels were normalized for the melanin content (ρ = 0.71; *p* < 0.001). A hair PhIP level of 10 ng/g melanin corresponded to an average PhIP intake of 0.5–1.0 µg per day [31,32]. However, the hair PhIP dosimeter was primarily derived from Whites and Asian/Pacific Islanders, which may limit its applicability for estimating PhIP intake in healthy AA men with highly pigmented melanin hair on free-choice diets.

The melanin content in hair varies significantly between individuals based on genetic factors, including ethnicity, age, and hair color. Darker hair has a higher concentration of eumelanin (black or brown pigment), while lighter hair contains less melanin or more pheomelanin (yellow or red pigment) [43]. AA men have significantly greater amounts of eumelanin in their hair than White men (Table 2, Figure 6B) [42]. This variability can lead to differences in how some chemicals bind to hair, making direct comparisons of exposure levels between individuals with different hair types challenging. Moreover, men of European ancestry often have higher hair density (more follicles per square centimeter) than men of African ancestry, resulting in more individual hair strands per scalp for White men [44,45]. African hair typically exhibits a more variable and narrower diameter along the hair shaft than the hair of Europeans [42]. How these morphological features influence PhIP accumulation in hair remains unclear. Thus, the large differences in hair melanin content and the differences in hair follicular density and shaft diameters between genetic ancestries may affect PhIP accumulation in hair and complicate the interpretation of hair PhIP levels and exposure assessments between AA and White men.

Basic drugs that are positively charged at physiological pH in the blood may exhibit increased binding to melanin due to electrostatic interactions with the carboxylic acid groups of melanin, while neutral drugs would be less affected by the extent of hair pigmentation [27]. While this conclusion may hold true for specific compounds, the affinity of PhIP for black fur in C57BL/6 mice is considerably greater than that observed for amino-α-carboline (AαC) and 2-amino-3,8-dimethylimidazo[4,5-*f*]quinoxaline (MeIQx), two other prevalent HAAs formed in cooked meats [30]. All three HAAs are weak bases and are predominantly protonated at physiological pH; however, neither AαC nor MeIQx have been detected in human hair thus far. Therefore, additional interactions between PhIP and eumelanin or other hair components may play a role in PhIP’s high binding affinity for dark-pigmented hair.

Other biological and lifestyle factors may impact hair PhIP levels. Hepatic cytochrome P4501A2 (CYP1A2) is the primary CYP enzyme involved in PhIP metabolism in humans [46]. In our previous work, we initially hypothesized that non-smoking individuals in our semi-controlled feeding studies with rapid CYP1A2 phenotype would have lower levels of unmetabolized PhIP in the bloodstream reaching the hair follicle and, therefore, lower PhIP levels in their hair. However, the hair PhIP levels were not influenced by (CYP1A2) activity, as assessed by the caffeine test, or the metabolic ratio of the major urinary CYP1A2 derived metabolite *N*^2^-(β-1-glucosiduronyl-2-(hydroxyamino)-1-methyl-6-phenylimidazo[4,5-*b*]pyridine to unmetabolized PhIP [31,32].

CYP1A2 expression is induced by the aryl hydrocarbon receptor (AHR) through a complex signaling cascade [47]. Its expression varies due to environmental factors, such as exposure to tobacco smoke, certain drugs, and components of the diet, including grilled meat, cruciferous vegetables, and coffee, which interact with AHR and induce CYP1A2 expression [48]. This induction can impact metabolism and the biological effects of PhIP and other procarcinogens bioactivated by CYP1A2. Smoking 10 cigarettes per day increases CYP1A2 activity by about 1.5-fold [49], and frequent consumption of well-done fried meat high in HAA content but low in polycyclic aromatic hydrocarbons also increases CYP1A2 activity by 1.5-fold [50]. In our previous studies, the range of CYP1A2 activity in the cohorts varied by 18-fold and 22-fold [31,32]. However, there was significant within-individual variation in the CYP1A2 phenotype and inconsistent responses to inducers of CYP1A2 over the course of the studies, which may have obscured a potential relationship between the caffeine-based CYP1A2 phenotype and PhIP levels accumulated in hair [51]. Furthermore, other dietary components, such as chlorophyllin and fibers, are reported to bind HAAs, potentially reducing their bioavailability and lowering hair PhIP levels [52,53]. These factors should be evaluated in future studies.

This pilot study did not measure the CYP1A2 phenotype in the AA or White cohorts. Given that volunteers were on free-choice diets, individual CYP1A2 phenotypes likely varied over time. The impact of smoking and alcohol consumption on hair PhIP levels for AA and NHW men are reported in Supplementary Table S3. Thirty-eight percent of AA men were current smokers (averaging 10 cig per day, 95% CI: 6.2, 13.8). Hair PhIP levels in this subgroup of smokers were significantly lower than those in AA men who were never or former smokers (median: 99.7 vs. 184.7 pg/g hair, *p* = 0.03; 6.5 vs. 10.9 ng/g melanin, *p* = 0.02), suggesting that cigarette smoking altered the PhIP pharmacokinetics. This effect could occur through the induction of hepatic CYP1A2 protein expression by tobacco smoke, leading to more metabolism and reduced PhIP levels in the blood following first-pass metabolism and, consequently, lower PhIP accumulation in hair. One study reported the formation of PhIP in mainstream tobacco smoke condensate at an average level of 16.4 ng per cigarette [54]; however, this finding remains controversial as no subsequent studies have confirmed PhIP in cigarettes. Additionally, specific liquid chromatography-mass spectrometry-based methods have not demonstrated an association between cigarette smoking and urinary PhIP levels [18,55,56]. Only two volunteers in the White cohort were current smokers, limiting any interpretation of smoking status on PhIP hair levels.

Thirty-one percent of AA men were current alcohol drinkers compared to 75% of the White men (*p* < 0.001) (Supplementary Table S3). While CYP2E1 is the primary CYP isoform involved in ethanol metabolism [57], it does not contribute to PhIP metabolism [25]. Therefore, alcohol consumption would not be expected to directly affect PhIP metabolism and hair levels. However, in this pilot study, the PhIP levels were significantly higher in AA drinkers than non-drinkers when reported as PhIP pg/g hair (median: 189.9 vs. 105.6, *p* = 0.013) but not when adjusted for hair melanin content. In the White cohort, hair PhIP levels did not differ significantly between drinkers and non-drinkers. Drinking status was further categorized as non-drinking, drinking alcohol less than weekly, or drinking weekly or more (Supplementary Table S4). AA men who drank weekly or more had significantly higher PhIP levels in hair than the non-drinkers and those drinking less than weekly (*p* = 0.028). However, these differences were not significant after normalizing for melanin content. In contrast, neither absolute hair PhIP levels nor melanin-adjusted PhIP levels showed statistically significant differences across drinking status groups in White men. It remains possible that alcohol consumption influences dietary preferences, such as increased meat intake, which could elevate PhIP exposure. A larger cohort is needed to evaluate the potential interplay between alcohol consumption, well-done meat diets, and hair PhIP levels.

**Figure 7:**
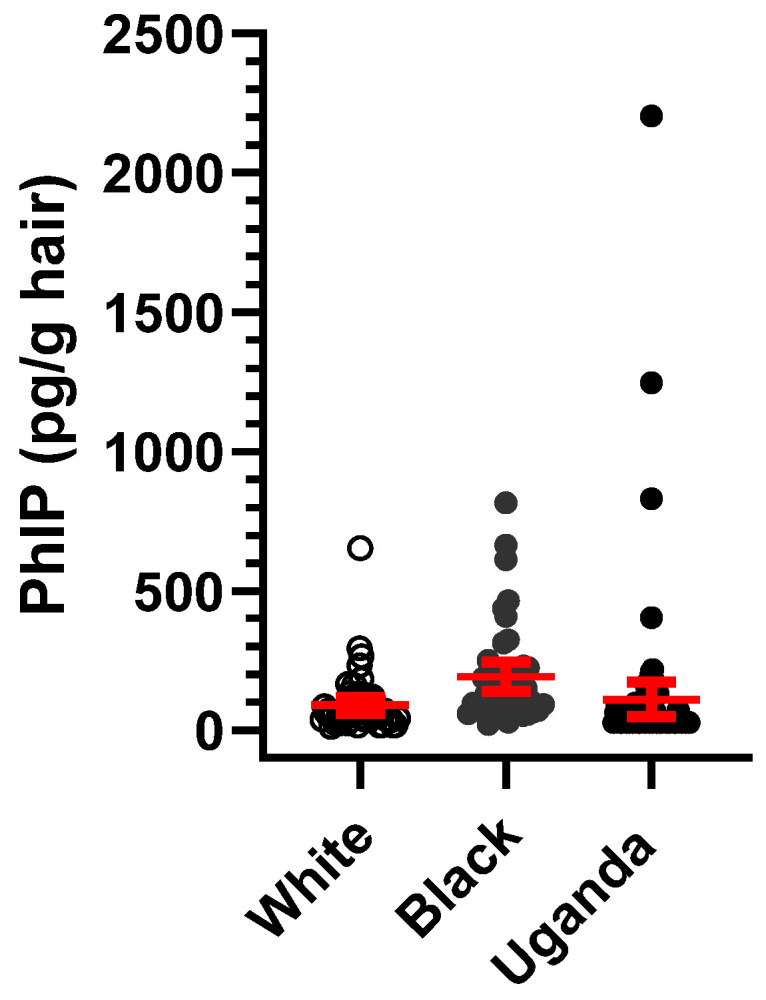
Scatter plots showing the mean and 95% CI for hair PhIP levels in White and African American men, and the Ugandan cohort.

We also employed the hair PhIP dosimeter in a pilot study conducted in southern Uganda, Africa (PADRE study), which investigated associations between polycyclic aromatic hydrocarbon exposure and esophageal cancer [58]. Both men and women were recruited in that prior study. Newly grown occipital scalp hair was collected and analyzed for PhIP. Of the 83 participants, 61 were men, and 22 were women: 25 men (41.0%) and 4 women (18.2%) had hair PhIP levels above the LOQ (Figure 7). Although the frequency of participants positive for PhIP was lower than in our AA cohort in Minneapolis, some participants from Uganda had 5 to 10 times higher hair PhIP levels than those observed in the Minneapolis cohorts. These preliminary findings suggest a subgroup of Africans consume significantly higher amounts of PhIP compared to AA and White men in the U.S. However, data on HAA levels in cooked meats in Africa are limited [59], underscoring the need for further exposure assessment on this potential prostate-cancer-causing agent in African cohorts, which also have elevated PC incidence [15,16].

Our study has several strengths, including being the first to investigate hair PhIP levels in healthy AA and Caucasian men on a free-choice diet in the U.S., with 100% detection of PhIP in the hair of AA participants. The analytical method to measure PhIP is highly sensitive, requiring only 20–25 mg hair per assay. Limitations include the collection of hair specimens at different times and settings, and some differences in the FFQs used to capture meat intake and cooking preferences between the cohorts. The conclusions drawn from this pilot study may differ in larger cohorts. Since the meat consumption data from the past seven days were gathered through self-reported surveys, recall bias may have affected the accuracy of the frequency of meat consumption. Furthermore, non-probability sampling methods were used to recruit AA and non-Hispanic White participants, which may have introduced sample bias of the participants’ characteristics (such as lifestyles and behavioral factors) and limit the applicability of findings to broader populations.

## 4. Conclusions

PhIP is frequently detected in the hair of omnivores. The substantial variation in melanin content between racial groups may influence PhIP binding to hair, complicating the adjustment of hair PhIP levels for melanin and potentially leading to inaccurate estimates of dietary PhIP intake between AA and White cohorts. Other differences in the morphologic features of hair among racial groups could also impact PhIP accumulation in hair. A comprehensive exposure assessment should report PhIP levels both per unit weight of hair and adjusted for melanin content. Notably, subsets of White, AA, and indigenous African men exhibit relatively high levels of PhIP in their hair, suggesting more frequent consumption of well-done cooked meat. Future studies are needed to evaluate high hair PhIP levels as a proxy for well-done cooked meat consumption in healthy White, AA, and indigenous African men and newly diagnosed PC patients, to investigate associations with aggressive PC pathology [6,7,8,9,33]. While the hair PhIP dosimeter measures exposure, it does not reflect the biologically effective dose. Combining other biomarkers, such as protein or DNA adducts formed with the reactive genotoxic intermediates, alongside the hair PhIP dosimeter, can provide a more comprehensive assessment of human health risks associated with this potential human prostate carcinogen [25,26].

## Figures and Tables

**Table 1:** Demographics on African American and non-Hispanic White male cohorts.

Characteristics, (N = 97)	African American (N = 45)	Non-Hispanic White (N = 52)	*p*-Value ^a^
Age at survey, median (IQR)	47.0 (30.0)	60.0 (12.5)	0.032
Body mass index(kg/m^2^), median (IQR)	28.2 (8.5)	27.5 (8.1)	0.34
Missing	0	5	
Smoking status, N (%)			<0.001
Current smoker	17 (38)	2 (3.8)	
Former smoker	12 (27)	17 (33)	
Never smoker	16 (36)	33 (63)	
Alcohol use, N (%)			<0.001
Current drinker	14 (31)	38 (75)	
Former drinker	25 (56)	8 (16)	
Never drinker	6 (13)	5 (9.8)	
Missing	0	1	

^a^ Wilcoxon rank sum test; Pearson’s Chi-squared test; Fisher’s exact test.

**Table 2:** Summary of PhIP levels, melanin content, and PhIP Levels adjusted for melanin for African American and non-Hispanic White male cohorts.

Characteristics, (N = 97) Median (IQR)	African American (N = 45)	Non-Hispanic White (N = 52)	*p*-Value ^a^
PhIP levels (pg/g hair)	144.0 (133.8)	58.1 (76.8)	<0.001
Melanin (mg/g)	16.1 (12.2)	8.3 (3.7)	<0.001
Adjusted PhIP levels (ng/g melanin)	7.9 (8.4)	7.7 (9.1)	0.53

^a^ Wilcoxon rank sum test.

## Data Availability

The data presented in this study are available on request from the corresponding author.

## Supplementary Materials

The following supporting information can be downloaded at: https://www.mdpi.com/article/10.3390/toxics13010042/s1, Table S1. Summary of meat consumption and cooking preferences for African American and non-Hispanic White men; Table S2. Correlations of charred barbecued meat consumption, PhIP levels, melanin content, PhIP levels adjusted for melanin for African American and non-Hispanic White men; Table S3. Summary of PhIP levels, melanin content, PhIP levels adjusted for melanin by smoking and alcohol drinking status for African American and non-Hispanic White men; Table S4. Summary of PhIP levels, melanin content, PhIP levels adjusted for melanin by alcohol drinking status for African American and non-Hispanic White men.
