# Supplementary material for: Biomonitoring PhIP, a Potential Prostatic Carcinogen, in the Hair of Healthy Men of African and European Ancestry
Source: Toxics. 2025 Jan 8;13(1):42. doi: 10.3390/toxics13010042 (PMC11769170; doi:10.3390/toxics13010042)
Supplement: Supplementary file 1 [file toxics-13-00042-s001.zip › toxics-3366317-supplementary.pdf]

Supplementary Material

Biomonitoring PhIP, a Potential Prostatic Carcinogen, in the Hair of Healthy Men of African and European Ancestry

Robert J. Turesky[1,*], Clarence Jones[2], Jingshu Guo[1,3], Kari Cammerrer[1] Laura A. Maertens[4], Emmanuel S. Antonarakis[5], Zhanni Lu[6], and Logan G. Spector[6,*]

[1] Masonic Cancer Center and Department of Medicinal Chemistry, University of Minnesota, Minneapolis, MN 55455, U.S.; rturesky@umn.edu (R.J.T); camme004@umn.edu (K.C)

[2] Hue-Man Partnership, 2400 Park Ave., Minneapolis, MN 55404, U.S.; clarencejones7428@gmail.com (C.J.)

[3] Clinical Research and Toxicology, Chromatography and Mass Spectrometry Division, Thermo Fisher Scientific, 355 River Oaks Parkway, San Jose, CA 95134, U.S.; jingshu.guo@thermofisher.com (J.G.)

[4] Masonic Cancer Center and Division of Environmental Health Sciences, School of Public Health, University of Minnesota, Minneapolis, MN, 55455, U.S.; maert006@umn.edu (L.A.M)

[5] Department of Medicine, University of Minnesota, Minneapolis, MN, 55455, U.S., anton401@umn.edu (E.S.A.)

[6] Masonic Cancer Center and Division of Pediatric Epidemiology and Clinical Research, University of Minnesota, Minneapolis, MN 55455, U.S.; luxxx371@umn.edu (Z.L.); spect012@umn.edu (L.G.S.)

* Correspondence: rturesky@umn.edu (R.J.T.); spect012@umn.edu (L.G.S.)

Table of Contents

Table S1. Summary of Meat Consumption and Cooking Preferences for African American and Non-Hispanic White Men..3

Table S2. Correlations of Charred Barbecued Meat Consumption, PhIP Levels, Melanin Content, PhIP Levels Adjusted for Melanin for African American and Non-Hispanic White Men 5

Table S3. Summary of PhIP Levels, Melanin Content, PhIP Levels Adjusted for Melanin by Smoking and Drinking Status for African American and Non-Hispanic White Men 6

Table S4. Summary of PhIP Levels, Melanin Content, PhIP Levels Adjusted for Melanin by Drinking Status for African American and Non-Hispanic White Men 7

Table S1. Summary of Meat Consumption and Cooking Preferences for African American and Non-Hispanic White Men

Characteristics (N = 97)	African American (N = 45)	Non-Hispanic White (N = 52)	p-value[a]
**In the past 7 days, how many times did you eat meat or poultry that was cooked charred/barbecued? Median (IQR)**	3.0 (4.0)	0.0 (0.0)	<0.001
**Any charred barbecued meat or poultry, N (%)**			<0.001
None	11 (24)	41 (79)	
Any	34 (76)	11 (21)	
**Frequencies of charred barbecued meat or poultry consumption, N (%)**			<0.001
None	11 (24)	41 (79)	
1-4 times	20 (44)	0 (0)	
5-7 times	10 (22)	11 (21)	
8-22 times	4 (8.9)	0 (0)	
**In the past 7 days, how many times did you eat seafood that was cooked charred/barbecued? Median (IQR)**	0.0 (1.0)	0.0 (0.0)	<0.001
Any charred barbecued seafood, n (%)			<0.001
None	31 (69)	50 (96)	
Any	14 (31)	2 (3.8)	
**Frequencies of charred barbecued seafood consumption, N (%)**			<0.001
None	31 (69)	50 (96)	
1-4 times	14 (31)	1 (1.9)	
5-7 times	0 (0)	0 (0)	
8-22 times	0 (0)	1 (1.9)	
**In the past 7 days, how many times did you eat processed meat that was cooked charred/barbecued? Median (IQR)**	0.0 (1.0)	0.0 (0.0)	<0.001
Any charred barbecued processed meat, n (%)			<0.001
None	25 (56)	49 (94)	
Any	20 (44)	3 (5.8)	
**Frequencies of charred barbecued processed meat consumption, N (%)**			<0.001

Characteristics (N = 97)	African American (N = 45)	Non-Hispanic White (N = 52)	p-value[a]
None	25 (56)	49 (94)	
1-4 times	18 (40)	3 (5.8)	
5-7 times	2 (4.4)	0 (0)	
8-22 times	0 (0)	0 (0)	
**Charred Barbecued Types, N (%)**			<0.001
0	11 (24)	39 (75)	
1	12 (27)	10 (19)	
2	10 (22)	3 (5.8)	
3	12 (27)	0 (0)	
**Any charred barbecued meat or poultry, seafood, processed meat, n (%)**			<0.001
None	11 (24)	39 (75)	
Any	34 (76)	13 (25)	
**Frequencies charred barbecued meat or poultry, seafood, processed meat, n (%)**			<0.001
None	11 (24)	39 (75)	
1-4 times	12 (27)	2 (3.8)	
5-7 times	11 (24)	10 (19)	
8-22 times	11 (24)	1 (1.9)	
**Charred barbequed food index[b], Median (IQR)**	4.0 (6.0)	0.0 (0.3)	<0.001

[a.] Wilcoxon rank sum test; Pearson’s Chi-squared test; Fisher’s exact test.
[b.] Derived from the sum of the frequencies of charred barbecued red meat or poultry, seafood, processed meat consumption.

Table S2. Correlations of Charred Barbecued Meat Consumption, PhIP Levels, Melanin Content, PhIP Levels Adjusted for Melanin for African American and Non-Hispanic White Men

Meat consumption and PhIP biomarkers	African American (N = 45)						Non-Hispanic White (N = 52)					
	Red meat/ poultry	Seafood	Processed meat	Charred barbecued food index	PhIP levels (pg/g hair)	PhIP levels (ng/g melanin)	Red meat/ poultry	Seafood	Processed meat	Charred barbecued food index	PhIP levels (pg/g hair)	PhIP levels (ng/g melanin)
**Red meat/poultry**	-	.489***	.483***	.949***	.012	.053	-	.146	.276	.933***	-.071	-.074
**Seafood**	-	-	.533***	.636***	**.365***	**.398****	-	-	-.049	.345*	-.272	-.192
**Processed meat**	-	-	-	.662***	.034	.047	-	-	-	.453***	.047	.096
**Charred barbecued food index** [a]	-	-	-	-	.033	.090	-	-	-	-	-.144	-.119
**PhIP levels (pg/g hair)**	-	-	-	-	-	.809***	-	-	-	-	-	.918***
**PhIP levels (ng/g melanin)**	-	-	-	-	-	-	-	-	-	-	-	-

Notes: Spearman Correlations.
Table S2 Interpretation: The table illustrated the rank-order correlation coefficient values in each cell between two variables of the six meat consumption or PhIP biomarkers that were listed along both the rows and columns within the African Americans and Non-Hispanic White groups of the table. The correlation coefficient values (range: -1 to 1) and negative or postive sign represented the strength and direction of the relationship between the variable in that row and the variable in that column: 0 to 0.1 (or 0 to -0.1): No correlation or very weak positive/negative correlation; 0.1 to 0.3 (or -0.1 to -0.3): Weak positive/negative correlation; 0.3 to 0.5 (or -0.3 to -0.5): Moderate positive/negative correlation; 0.5 to 0.7 (or -0.5 to -0.7): strong positive/negative correlation; 0.7 to 1 (or -0.7 to -1): very strong positive/negative correlation.
[a.] Derived from the sum of the frequencies of charred barbecued red meat or poultry, seafood, processed meat consumption.
- : indicated that either the correlation tests were not applicable (i.e. correlations between the same variables of meat consumption or PhIP biomarkers) or the correlation tests had been conducted previously and thus the test results did not display twice in the table.
*: $p < .05$. **: $p < .01$. ***: $p < .001$

Table S3. Summary of PhIP Levels, Melanin Content, PhIP Levels Adjusted for Melanin by Smoking and Alcohol Drinking Status for African American and Non-Hispanic White Men

Median (IQR)	African American (N = 45)						Non-Hispanic White (N = 52)					
	Current smokers N = 17	Non-smokers N = 28	P [a]	Current drinkers N = 14	Non-drinkers N = 31	P [a]	Current smokers N = 2	Non-smokers N = 50	P [a]	Current drinkers N = 38	Non-drinkers N = 13	P [a]
**PhIP levels (pg/g hair)**	99.7 (73.2)	184.7 (173.1)	0.033	189.9 (148.6)	105.6 (123.1)	0.013	20.6 (3.8)	59.3 (77.8)	0.043	57. 9 (78.9)	58.1 (45.6)	0.45
**Melanin (mg/g)**	14.9 (14.8)	16.9 (9.1)	0.79	20.0 (11.2)	15.4 (11.8)	0.11	10.8 (2.2)	8.3 (3.5)	0.26	8. 6 (3.7)	7.4 (2.6)	0.17
**PhIP levels (ng/g melanin)**	6.5 (3.5)	10.9 (9.3)	0.02	12.5 (8.3)	6.8 (6.6)	0.13	1.9 (0.0)	8.3 (9.0)	0.024	8.4 (9.5)	6. 5 (9.0)	0.60

[a] Wilcoxon rank sum test.

Table S4. Summary of PhIP Levels, Melanin Content, PhIP Levels Adjusted for Melanin by Alcohol Drinking Status for African American and Non-Hispanic White Men

Median (IQR)	African American (N = 45)				Non-Hispanic White (N = 52)			
	Not drinking N = 31	Less than drinking weekly N = 11	Drinking Weekly or more N = 3	P [a]	Not drinking, N = 13	Less than drinking weekly N = 7	Drinking Weekly or more N = 31	P [a]
**PhIP levels (pg/g hair)**	105.6 (123.1)	158.4 (74.8)	313.8 (50.2)	0.028	58.1 (45.6)	98.0 (70.5)	57.6 (72.1)	0.23
**Melanin (mg/g)**	15.4 (11.8)	18.9 (13.2)	22.5 (6.5)	0.27	7.4 (2.6)	7.3 (4.5)	8.6 (3.4)	0.36
**PhIP levels (ng/g melanin)**	6.8 (6.6)	10.5 (9.4)	14.4 (2.2)	0.15	6.5 (9.0)	16.0 (11.9)	6.6 (7.9)	0.17

[a] Kruskal-Wallis rank sum test.
